# Occurrence and Public Health Implications of *Brucella Abortus* and Antimicrobial Residues in Raw Cow Milk in Bukombe District, Tanzania

**DOI:** 10.1002/vms3.70944

**Published:** 2026-04-09

**Authors:** Makoye Mhozya, Hezron Emmanuel Nonga

**Affiliations:** ^1^ College of Agriculture Mwalimu Julius K. Nyerere University of Agriculture and Technology Musoma Tanzania; ^2^ Department of Veterinary Medicine and Public Health College of Veterinary Medicine and Biomedical Sciences Sokoine University of Agriculture Morogoro Tanzania

**Keywords:** antibiotics, brucellosis, seroprevalence, withdrawal period

## Abstract

**Background:**

Milk produced from indigenous cattle in Tanzania is always contaminated with biological and chemical hazards, a situation that puts the public at risk of health threats.

**Objectives:**

A cross‐sectional study was conducted in October 2016 and January 2017 to estimate the seroprevalence of brucellosis and associated risk factors for infection and establish the status of antimicrobial residues in raw cow milk in Bukombe district.

**Methods:**

A total of 221 blood and milk samples from lactating cows were analysed for *B. abortus* using serological and diagnostic PCR tests, and antimicrobial residues tested by Delvo and HPLC. Alongside, 55 farmers were interviewed to explore the risk factors for *Brucella* infection and antimicrobial uses in cattle.

**Results:**

Cattle and herd seroprevalence of brucellosis was 1.4% and 3.8 respectively involving *B. abortus*. Up to 11.6% of the milk samples had tetracycline residues at mean concentration of 19.1 ± 17.6 µg/L. The mean concentration of OTC and TTC residues were 8.5 ± 4.8 µg/L and 10.6 ± 17.5 µg/L, respectively. The reported cow abortions, feeding dogs with placenta and aborted foetus, communal grazing and watering points, introduction of new animals in the herd and grazing in wildlife areas had ≥ 40% of responses as factors for transmission of brucellosis. Indiscriminate uses of antibiotics were common because of easy accessibility from veterinary shops and open livestock markets.

**Conclusions:**

Brucellosis is prevalent in cattle and antimicrobial residues in raw milk is of public health concerns. To tackle the two public health problems, a coordinated One Health approach is recommended.

## Graphical abstract

Poor husbandry practices in Bukombe District accelerate diseases in livestock and indiscriminate uses of antimicrobials. Cow abortions, raw cow placenta and aborted fetuses feeding dogs, communal grazing and watering and introduction of new animals predict brucellosis in the herd. Farmers who use veterinary drugs with limited knowledge on withdrawal periods and the health effects of residues predict antimicrobial residues in milk. Seroprevalence of brucellosis in cattle was 1.4%, and tetracycline residue cow milk contamination rates were 11.6% with a mean concentration of 19.1 ± 17.6 µg/L. To tackle these public health threats needs a One Health approach.

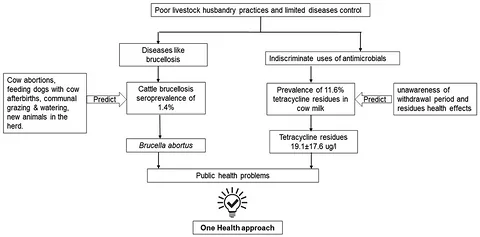

## 1 Introduction

Livestock sector in Tanzania grows at a rate of 3.4% and contribute to about 7.4% to the Gross Domestic Product (GDP). Of the 6.7% GDP contribution by livestock sector, 40% comes from meat, 30% from dairy industry and 30% from other livestock sources (TLMP 2017; MLF 2025). If well developed, the dairy industry has the potential to be a subsector that reduces poverty in Tanzania. More than 80% of the milk comes from indigenous cattle kept in rural areas raised under extensive grazing system characterised by poor livestock husbandry practices (National Livestock Policy 2006; TLMP 2017). The cattle population in Tanzania is estimated at 39.2 million (TLMP 2017; MLF 2025). Despite having the large livestock population in Tanzania; the performance of livestock industry is still poor possibly due to a number of challenges that include livestock diseases (National Livestock Policy 2006).

Apart from being nutritionally important food for humans, cow milk may be a potential source of biological and chemical hazards (Dey and Karim 2013; Bukuku et al. 2015). Milk‐borne diseases like brucellosis are rampant in humans, especially in developing countries partly because of consumption of raw unpasteurised milk (Mekuria et al. 2014; Bukuku et al. 2015; Mengele et al. 2023). Many cases of human brucellosis have been reported in Tanzania, and the disease causes non‐specific signs which are mistakenly diagnosed as malaria, typhoid fever, and rheumatic fever (Crump et al. 2013; Cash‐Goldwasser et al. 2018; Bodenham et al. 2020). Contacts between humans and infected animals are among the common means of transmission. Consumption of raw unpasteurised milk, raw meat, blood, and other food of animal origin are drivers to the transmission of brucellosis from livestock to humans (Tumwine et al. 2015; Adesokan et al. 2016; Tadesse 2016; Cash‐Goldwasser et al. 2018). A study by Mellau et al. (2009) reported the increase of human brucellosis from 35.6% in 2004 to 58.1% in 2005 in livestock‐wildlife interface in Serengeti ecosystem inhabited by pastoralists whose livestock are in constant contacts with wildlife. Studies in Tanzania show that the sero‐prevalence of brucellosis in cattle ranges between 2% and 90% (Weinhäupl et al. 2000; Swai et al. 2005; Karimuribo et al. 2007; Chitupila et al. 2015; Assenga et al. 2015; Mengele et al. 2023). The factors for persistence of brucellosis in livestock are based on the grazing system, lack of test and slaughter policy, and practical disease control programmes (Assenga et al. 2015; Mengele et al. 2023).

The uses of antimicrobials in farm animals are a common practice in Tanzania for treatment, prophylaxis and growth promotion (Katakweba et al. 2012). The average annual utilisation of antimicrobials in animals in Tanzania is more than 1.6 million kilograms (Sangeda et al. 2021). Nevertheless, indiscriminate uses of antimicrobials in farm animals goes along with farmers not complying with drug withdrawal periods before harvesting the food of animal origin (Mdegela et al. 2021; Sangeda et al. 2021). According to reports, Tanzanian raw cow milk has antimicrobial residues as high as 766.3 µg/L (Ramadhani 2015). Food and Agricultural Organization and World Health Organization (FAO/WHO) and the European Union (EU) have recommended a maximum residue limit (MRL) of 100 µg/L for tetracycline, oxytetracycline and/or chlortetracycline in milk (EU 2009; CAC 2023).

Based on the previous studies, brucellosis, a priority zoonosis and major public health problem in Tanzania, is reported in cattle in different areas of the country but not in the Bukombe district. Likewise, antimicrobial residues that cause antimicrobial resistance, an emerging global public health threat, are rarely reported in indigenous cattle that produces more than 80% of the milk consumed in Tanzania. Therefore, this study explored seroprevalence of brucellosis in cattle and established the contamination rates of antimicrobial residues in cattle of Bukombe district, Tanzania.

## 2 Materials and Methods

### 2.1 Study Area, Animals and Design

Bukombe district is one of the five districts in Geita region whose geographical coordinates are 3°28′0″S, 31°54′0″E. It has a human population of 407,102 and a cattle population of 253,100 (BDC Report 2020). The district has 17 administrative wards, but most livestock keepers are found in 9 wards namely Namonge, Ng'anzo, Busonzo, Butinzya, Bulega, Lyambamgongo, Bugelenga, Bukombe and Iyogelo. Most of cattle are Tanzania Short Horned Zebu (TSHZ) and Ankole breeds which are kept by agropastoralists which rarely get veterinary services and other disease control programmes. The study design was cross‐sectional which was conducted in October 2016 and January 2017.

### 2.2 Selection of Study Farms and Animals

A total of 17 villages from 9 wards with livestock keepers were purposively selected for study based on having a big number of livestock keepers. The smallholder farmers in Bukombe District were obtained by a random selection procedure from households with lactating cattle at each study village. The Ward Livestock Field Officer (WLFO) helped to prepare the list of households with lactating cows that were used as sampling frames. A total of 54 cattle herds were selected for study. The sample size of 221 lactating cattle was selected to allow a detection level of 5% with 99% certainty (Canon and Roe 1986).

### 2.3 Questionnaire Survey

The developed questionnaire consisted of four sections that addressed the (i) socio‐demographic information, (ii) cattle management and diseases control (iii) possible factors for brucellosis infection in cattle and, (iv) uses of antibiotics in cattle and farmers awareness on the drug withdrawal period. The questionnaire was administered in Kiswahili, the national language in Tanzania. Further, each participant was interviewed separately to improve participation, and all interviews were organised privately. Prior to survey, the questionnaire was pre‐tested to 10 agropastoralists from a village outside the study area to assess clarity, relevance, and logical flow.

### 2.4 Blood and Milk Sampling Procedures and Handling

Cow blood sample was collected from the jugular vein into plain vacutainer tubes and after clotting, it was centrifuged at 1500 speeds for 10 min to obtain serum which was aliquoted into cryovials. Milk sample was collected from the udder into sterile 15 mL falcon tube. All samples were stored in cool boxes packed with ice parks during field work and subsequently stored in a freezer at –21°C until analysis. Sample analysis was done at laboratories of Sokoine University of Agriculture (SUA) and Tanzania Medicines and Medical Devices Authority (TMDA).

### 2.5 Analysis of Serum Samples

Out of 221 sera samples collected, 219 were tested for antibody against *Brucella* spp. by the Rose Bengal Plate test (RBPT) as a screening test and confirmed by using competitive enzyme linked immunosorbent assay (c‐ELISA) (Nielsen and Ewalt 2010; OIE 2012). Animals that were positive on c‐ELISA were considered as *Brucella* seropositive. A herd was regarded as seropositive for brucellosis when at least one animal reacted positive for c‐ELISA.

### 2.6 Analysis of *B. abortus* in Milk by PCR

Three milk samples that were from *Brucella* seropositive cows were analysed for *bcsp31* gene of *B. abortus* as described by Navarro et al. (2002). The DNA was extracted kit (Genomic DNA Purification Kit, Thermo Scientific USA) as described by the manufacturer. DNA quantification was done with Nano‐drop Thermo (Ferment as USA) and working aliquots of all extracted DNA samples were adjusted at same concentration level, that is, 50 ng/L. The B4/B5 primers (Bioline, Inc., Taunton, MA, USA) were used to amplify 223‐bp fragment that contained the target gene for *B. abortus*. The forward primer sequence used was 5^′^ TGG CTC GGT TGC CAA TAT CAA 3 and the reverse primer 5^′^ CGC GCT TGC CTT TCA GGT CTG 3^′^. DNA from *B. abortus* reference strain was used as a positive control and a negative control contained all the components of the reaction mixture except DNA. After amplification, 10 µL of the amplicon was analysed on 0.8% agarose gel.

### 2.7 Qualitative Analysis of Antimicrobial Residues in Milk by Delvo SP Test

A total of 198 out of 221 collected milk samples were analysed by Delvo SP test kit (Delft, Netherlands) that used *Bacillus stearothermophilus var. calidolactis* as the test organism and the procedures were as described by the manufacturer. Delvo SP test kit detection limit varies depending on the type of antimicrobial residues present, but the test is designed to detect antibiotics at or below their Maximum Residue Limits (MRLs). A positive control of 0.1 mL gentamycin 10% (Laprovet, France) solution was used while fresh UHT milk bought from the shop was used as negative control.

### 2.8 Analysis of Antimicrobial Residues in Milk by Chromatographic Method

High Performance Liquid Chromatography (HPLC) was used to analyse for concentration of tetracyclines (chlortetracycline, tetracycline and oxytetracycline) in milk samples since its use in cattle was reported by all the farmers (*n* = 54). HPLC analysis involved 10 out of 23 milk samples that tested positive for antimicrobial residues by Delvo SP test because of cost limitations. The milk analysis by HPLC followed the Official Methods 995.09 (AOAC International 2000) as described by Ghidini et al. (2003) and USA: CLG‐TET2.04 (2011) with some modifications. The analytical details of HPLC are shown in the supplementary materials 1.

### 2.9 Data Analysis

Data were entered into Microsoft Excel spread sheet and analysed using Epi Info Version 7 (Centre for Disease Control, Atlanta, USA). Descriptive statistics (percentages, frequencies, medians and means) were used to describe the proportions of sociodemographic characteristics/farm, livestock husbandry practices, diseases and pest control, livestock extension services and uses of veterinary drugs. Logistic regression was used to assess the risk factors associated with transmission of brucellosis in cattle and occurrence of antimicrobial residues in raw cattle milk. Chi‐square (*χ* ^2^) and confidence intervals were used to compare proportions of categorical variables like risk factors for brucellosis; a probability of *p* < 0.05 was statistically significant. Examined factors for brucellosis included repeated abortion, giving raw placenta and aborted foetus to dogs, communal grazing and watering points, introduction of new animals in the herd and grazing in wildlife protected areas. Examined factors for antimicrobial residues in milk were buy veterinary drugs and treat, education on antimicrobials, knowledge effects of antimicrobials and withdrawal period.

Based on the c‐ELISA results, prevalence was presented at individual animal and farm (herd) levels. Agreement between RBPT and c‐ELISA as tests for diagnosis of bovine brucellosis was established by using Cohen's κ coefficient (κ). Interpretation of the κ value was done as follows: poor (κ = 0), slight (0.01 < 0.20), fair (0.21< κ < 0.40), moderate (0.41 < κ < 0.60), almost perfect (0.61< κ < 0.80), and excellent (0.81 < κ < 1.00) (Landis and Koch 1997).

## 3 Results

### 3.1 Demographic Characteristics of the Respondents

Table 1 summarises the results of demographic characteristics of the respondents. A total of 54 respondents were interviewed most of the respondents had primary education (51.9%; 95% CI = 87.7–100), were male (98.2%; 95% CI = 93.3–100), had the age below 40 years (88.8%; 95% CI = 92.6–100) and none of them had attended training on livestock husbandry. The dominant ethnic group was Sukuma (90.7%; 95% CI = 92.8–100), and all the respondents were agropastoralists.

**TABLE 1:** Demographic characteristics of the respondents and study cattle in the selected wards in Bukombe district (*n* = 54).

Demographic information	Category	Number (%) of respondents	95% CI
Sex	Male	53 (98.2)	93.3–100
	Female	1 (1.9)	0.05–9.9
Age	<40	6 (11.1)	4.2–22.6
	>40	48 (88.9)	92.6–100
Education	Informal	23 (42.6)	29.2–56.8
	Primary	28 (51.9)	37.8–65.7
	Secondary	1 (1.9)	0.1–9.9
	College	2 (3.7)	0.5–12.8
Ethnicity	Sukuma	51 (90.7)	84.6–98.8
	Sumbwa	2 (3.7)	0.5–12.8
	Ha	1 (1.9)	0.1–9.9
Occupation	Agropastoralists	54 (100)	93.4–100
Grazing system	Extensive	54 (100)	93.4–100
Feed supplements of concentrates	No	54 (100)	93.4–100
Diseases prevention measures	None	54 (100)	93.4–100
Control ticks and flies of cattle by use of acaricides	Yes	19 (35.2)	22.7–49.4
	No	35 (64.8)	50.6–77.3
Common diseases of cattle	East Coast Fever	46 (85.2)	72.9–93.4
	Trypanosomiasis	30 (55.6)	41.4–69.1
	Foot and mouth disease	19 (35.2)	22.7–49.4
	Contagious bovine pleuropneumonia	16 (29.6)	17.9–43.6
	Black quarter	11 (20.4)	10.6–33.5
	Helminthiasis	7 (13)	5.4–24.9
	Lumpy skin disease	1 (1.9)	0.05–9.9
Experience calf mortalities in the herd?	Yes	54 (100)	93.4–100
Biodata of the study cattle (*n* = 221)			
Breed	TSHZ	128 (57.9)	51.1–64.5
	Ankole	93 (42.1)	35.5–48.9
Age (years)	≤7	207 (93.7)	89.6–96.5
	>7	14 (6.3)	3.5–10.4
Herd size (*n* = 54)	≤50	36 (66.7)	52.5–78.9
	>50	18 (33.3)	21.1–47.5

Abbreviations: CI, confidence interval; TSHZ, Tanzania Short Horn Zebu.

### 3.2 Information About the Study Cattle and Management System

Table 1 further shows biodata of cattle and the management system. A total of 221 lactating cows were involved in the study majority were Tanzania Shorthorn Zebu (TSHZ) (57.9%) and their age was between 3 and 7 years (93.7%). The cattle herd size was less than 50 animals in most farms (66.7%) and all the cattle herds in the study villages were extensively grazed in communal grazing areas. No supplementary feed was given to the animals. A few farmers (35.2%) reported to occasionally apply acaricides to control ticks and flies. All the farmers reported to have no disease prevention programs in their cattle, and the veterinary services were not available. Vetor‐borne diseases in particularly East Coast Fever (85.2%) and trypanosomiasis (55.6%) were commonly reported. All farmers reported encountering some frequent calf mortalities in their herd.

### 3.3 Factors Considered to Facilitate Transmission of Brucellosis in Cattle

Table 2 shows nine factors which were perceived by the respondents to facilitate transmission of brucellosis in cattle. The factors like repeated abortion, giving raw placenta and aborted foetus to dogs, communal grazing and watering points and grazing in wildlife protected areas had ≥ 40% of responses as factors for transmission of brucellosis. Logistic regression was done to the factors considered as risk for transmission of brucellosis in cattle and were all non‐statistically significant.

**TABLE 2:** Factors for transmission of brucellosis in cattle (*n* = 54).

Factor	Number (%) of respondents	95% CI
Communal grazing and watering points	53 (98.1)	90.1–99.9
Giving raw placenta and aborted foetus to dogs	41 (75.9)	62.4–86.5
Presence of wild animals in the village grazing land	34 (62.9)	48.7–75.7
Grazing in wildlife protected areas during scarcity of pastures	31 (57.4)	43.2–70.8
Repeated abortion	29 (53.7)	39.6–67.4
Buying or selling of animals	27 (50.0)	36.1–63.9
Throwing of placenta and aborted foetus in the bush	9 (16.7)	7.9–29.3
Lack of diseases prevention measures	5 (9.3)	3.1–20.3
Sharing of breeding bulls between farms	3 (5.6)	1.2–15.4

### 3.4 Factors That May Lead to the Occurrence of Antimicrobial Residues in Milk

Questionnaire results indicated that all respondents (*n* = 54) admitted using antimicrobials in for treatment of different diseases of cattle. Tetracyclines were used by all farmers for different purposes. Other antimicrobials used were penicillin (98.1%; 95% CI = 90.1–99.5), sulphonamides (85.2%; 95% CI = 72.9–93.4) and tylosin (55.6%; 95% CI = 41.4–69.1). Other veterinary drugs that were commonly used were antitrypanosomes (isometamidium chloride and diminazene aceturate) and anthelmintics (albendazole, mebendazole, levamisole and ivermectin). The farmers reported to buy and administer the drugs to their animals. The veterinary drugs were being bought from veterinary shops and open livestock markets without restrictions. Few respondents (37%; 95% CI = 24.3–51.3) were aware of drug residues in the cow milk. Majority (79.6%; 95% CI = 66.5–89.4) of respondents knew that drug residues in milk have health effects to the milk consumers, but they did not know the effects. Only 40.6% (95% CI = 27.6–54.9) of the respondents reported to abide with the drug withdrawal periods. Logistic regression analysis was done to some of the factors considered as risk for occurrence of antimicrobial residues in raw milk and neither of those factors were statistically significant.

### 3.5 Sero‐Prevalence of Brucellosis in Cattle

Of the 219 sera samples screened for brucellosis by RBPT, three (1.4%; 95% CI = 0.3–2.9) were positive. Two positive serum samples were from Namonge ward and one from Ng'anzo ward (Table 3). The overall animal seroprevalence of brucellosis in cattle was 1.4% while the herd seroprevalence was 3.8%. There was an excellent (perfect) κ agreement between RBPT and c‐ELISA as tests diagnostic methods for brucellosis (κ = 1) as shown in Table 4.

**TABLE 3:** Distribution of Rose Bengal Plate Test results of cattle in different wards (*n* = 219).

Wards	Number of cattle tested	Positive serum samples (%)	Negative serum samples
Namonge	36	2 (5.6)	34
Ng'anzo	28	1 (3.6)	27
Busonzo	35	0 (0.0)	35
Butinzya	7	0 (0.0)	7
Bulega	24	0 (0.0)	24
Lyambamgongo	23	0 (0.0)	23
Bugelenga	24	0 (0.0)	24
Bukombe	18	0 (0.0)	18
Iyogelo	24	0 (0.0)	24
Total	219	3 (1.4)	216

**TABLE 4:** Overall seroprevalence of brucellosis in cattle of Bukombe district based on RBPT and c‐ELISA (*n* = 219).

	RBPT	c‐ELISA	Test agreement
Total	219	219	κ = 1*
Negative sera	216	216	
Positive sera	3	3	
Cattle seroprevalence	1.4%	1.4%	

^*^ The agreement between RBPT and c‐ELISA to detect *Brucella* infection was perfect (*κ* = 1).

### 3.6 Detection of DNA of *Brucella Abortus* in Raw Milk

**FIGURE 1:**
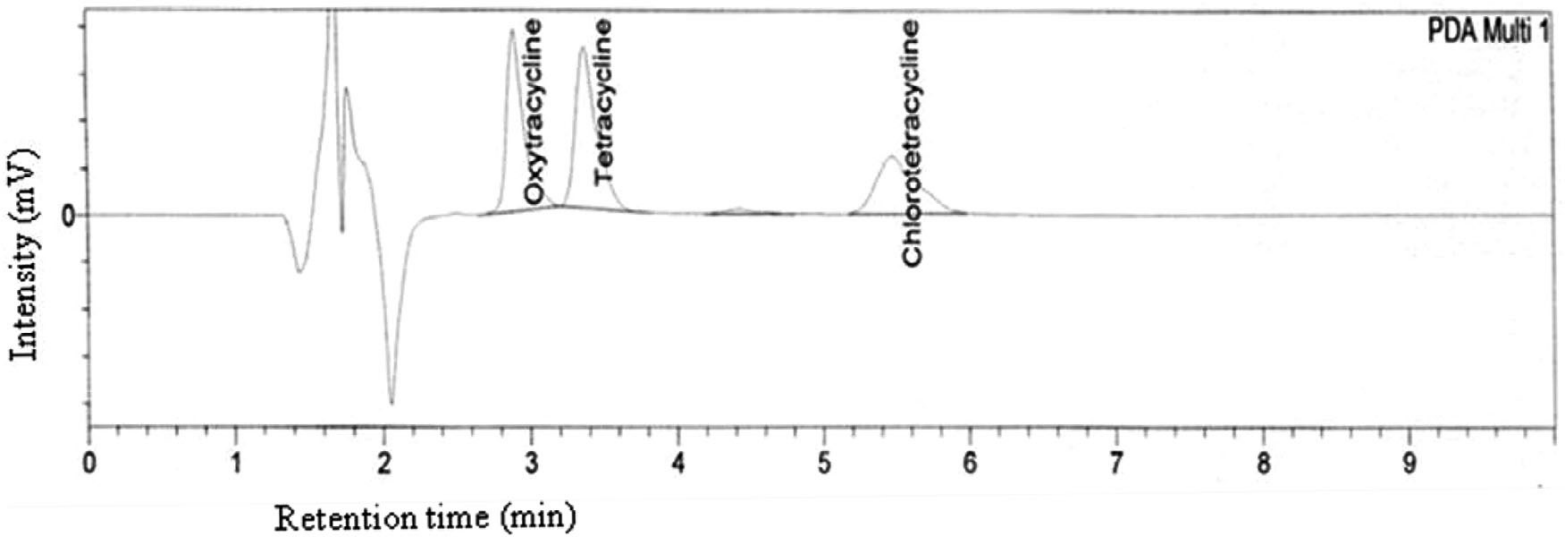
Agarose gel electrophoresis photograph showing amplification products of *B. abortus* from cow milk. The desired band size of *bcsp31* gene is at 223 bp. Lanes M (left and right) is a 50 kb molecular weight marker; lanes NC and PC are negative and positive control respectively. Note that lane 4 is positive amplicon while lanes 2 and 3 are negative milk samples.

Three milk samples (two from Namonge and 1 from Ng'anzo wards) from *Brucella* seropositive cows were analysed for presence of DNA of *B. abortus*. One sample from Namonge ward was positive with PCR since a band of 223 bp was observed (Figure 1).

### 3.7 Qualitative Results of Antimicrobial Results in Milk

A total of 198 milk samples were analysed for antimicrobial residues using Delvo test kits. It was found that 23 milk samples were positive which is equivalent to 11.6% (95% CI = 7.7–16.9) (Table 5). The herd prevalence of antimicrobial residues was 34.8% (95% CI = 7.5–16.9). In terms of wards, Busonzo had the highest number of milk samples with antimicrobial residues (29.4%) whereas Iyogelo had the lowest (4.3%).

**TABLE 5:** Delvo test results of 198 milk samples presented per ward.

Ward	Number of milk samples tested	Positive milk samples	Negative milk samples
Busonzo	17	5 (29.4)	12
Lyambamgongo	23	5 (21.7)	18
Bugelenga	26	4 (15.4)	22
Butinzya	7	1(14.3)	6
Ng'anzo	24	3 (12.5)	21
Bukombe	16	1 (6.3)	15
Namonge	39	2 (5.1)	37
Bulega	22	1 (4.5)	21
Iyogelo	24	1 (4.3)	23

### 3.8 Levels of Tetracycline Residues in Raw Milk

**FIGURE 2:**
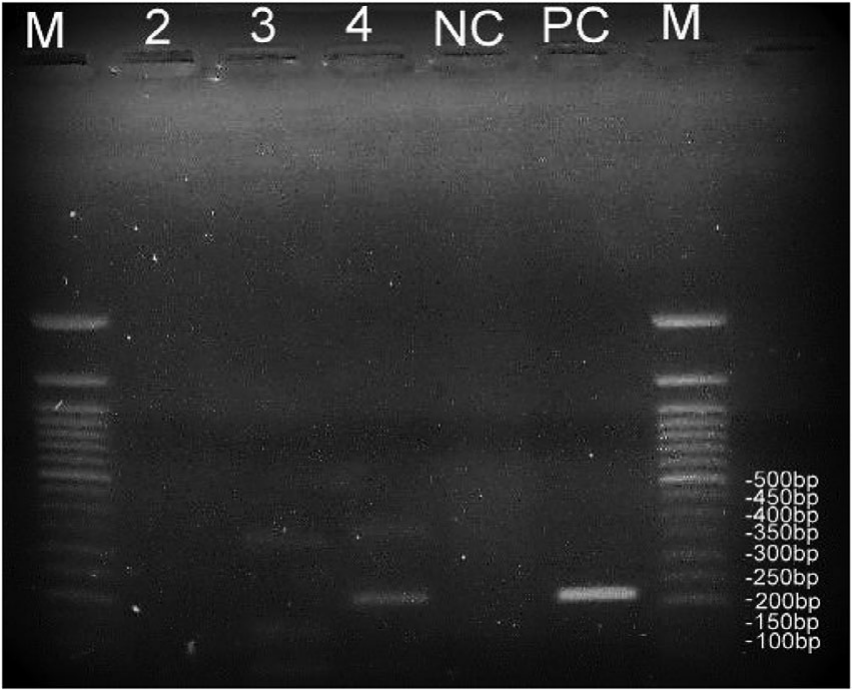
A chromatogram indicating tetracyclines residues in raw cow milk samples. Note that the mean concentrations of oxytetracycline residues was 8.5 ± 4.8 and that of tetracycline was 10.6 ± 17.5 µg/L.

Only 10 out of 23 positive milk samples by Delvo test kits were analysed for tetracycline residues by HPLC because of costs limitations. Among the selected 10 milk samples, 9 were confirmed and quantified to have at least one detectable type of tetracyclines by HPLC analysis (Table 6 and Figure 2). Neither of the quantified milk samples had measurable amounts of Chlortetracycline (CTC). The overall mean concentration of tetracyclines (TCs) in selected milk samples (*n* = 10) was 19.1 ± 17.6 µg/L. The mean concentration of oxytetracycline (OTC) and tetracycline (TTC) were 8.5 ± 4.8 µg/L and 10.6 ± 17.5 µg/L, respectively. Of importance, 70% of the quantified raw milk samples had OTC at varying concentrations and 40% of the quantified raw milk samples had TTC at varying concentrations. Comparing the observed values of TCs with the recommended MRL of 100 µg/L (CAC 2023) in milk of cattle, all the milk samples had TCs residues within acceptable levels.

**TABLE 6:** Concentration of tetracylines in selected raw milk samples (*n* = 10).

Sample ID	OTC (µg/L)	TTC (µg/L)	CTC (µg/L)	Total conc. (µg/L)
SM9	11.0	53.4	0.0	64.4
ED6	11.5	11.9	0.0	23.4
GF2	0.0	18.5	0.0	18.5
SM22	0.0	11.8	0.0	11.8
FD6	11.1	0.0	0.0	11.1
BD5	11.0	0.0	0.0	11.0
GA3	10.8	0.0	0.0	10.8
FD4	10.6	0.0	0.0	10.6
DB4	10.4	0.0	0.0	10.4
FF1	0.0	0.0	0.0	0.0
Mean conc. (µg/L)	8.5 ± 4.8	10.6 ± 17.5	0.0 ± 0	19.1 ± 17.6

*Note*: The recommended MRL for OTC, TTC and CTC is 100 µg/L (CAC 2023).

## 4 Discussion

The purpose of the current study was to estimate the seroprevalence of *B. abortus* infection in lactating cows, assess the risk factors for the infection and analyse for the antimicrobial residues in raw cow milk in Bukombe District. It was found that most of the livestock keepers were agropastoralists who kept local breeds cattle under extensive management. Feeding raw placenta and aborted foetus to dogs can increase the transmission of brucellosis among the domestic animals. Similar observation has been reported by Temba et al. (2019) in Mikumi‐Selous ecosystem whereby frequent abortions was associated with *Brucella* seropositivity in cattle. Education to farmers and public on how best brucellosis can be overcome in cattle is recommended.

The current study estimated a seroprevalence of brucellosis in cattle to be 1.4% while the herd prevalence was 3.8%; which are within the previous seroprevalence studies in Tanzania which range between 2% and 90% (Chitupila et al. 2015; Assenga et al. 2015; Temba et al. 2019). It is also within the ranges reported elsewhere in Africa (Hesterberg et al. 2008; Chota et al. 2016). The variation in animal sero‐prevalence of brucellosis could be contributed by variation in animal production systems in peri‐urban, diagnostic method used, urban and rural settings (Karimuribo et al. 2007; Lyimo 2013).

Further, one of the seropositive milk samples was confirmed to have a *Brucella bcsp31* which proves that traditional cattle of Bukombe district are exposed to *B. abortus*. This implies that the other two seropositive samples may be due to other species of *Brucella* like *B. mellitensis* and/or *B. ovis*. It is also possible that at the time of milk sampling, *B. abortus* wasn't shed into milk since its shedding is intermittent (Capparelli et al. 2009; Wareth et al. 2014). It was further observed that both RBPT and c‐ELISA serological tests gave similar results suggesting that sera with high RBT score may not require confirmation with other tests such as c‐ELISA. Therefore, RBPT can be routinely used for screening of *Brucella* infection since it is cost effective and suitable method under different conditions compared to others which need more sophisticated equipment in better laboratories.

The qualitative results of milk samples by Delvo test indicated that 11.6% of the tested samples had antimicrobial residues as previously reported by Ridhiwan (2015). At the herd level, it was found that 34.6% of the herds sampled had detectable levels of antimicrobial residues. This is a significant finding since the public is continuously exposed to low levels of antimicrobial residues which has public health implications including development of resistant bacteria. The observed results are higher than the studies by Kivaria et al. (2006) and Mdegela et al. (2009) but lower than the report by Rwehumbiza et al. (2012) and Karimuribo et al. (2015) in Tanzania. Elsewhere, there have been variable results on antimicrobial residues in milk. Lower levels than indicated in this study were reported by Addo et al. (2011) and Grădinaru et al. (2011) at the range of 3% to 5%. Higher levels than this study were reported elsewhere at the range of 24% to 50% (Salman et al. 2012; Aalipour et al. 2013; Mangsi et al. 2014; Ahlberg et al. 2016; Orwa et al. 2017). The variation in proportions of milk with antimicrobial residues is probably due to different production and monitoring systems in various livestock keepers in different countries but also the laboratory analysis used in testing of the antimicrobial residues. It can also be attributed to variation in levels of education, knowledge, strictness in observation of withdrawal periods by the farmers.

Interestingly, 90% of the Delvo test positive milk contained at least one or two types of tetracycline residues suggestive of rampant uses of these antibiotics in traditional cattle production in Bukombe. However, the mean levels of tetracyclines were 19.1 ± 17.6 µg/L which is below the recommended MRL of 100 µg/L for TCs in raw cow milk (Applegren et al. 1999; CAC 2023; EU 2009). Although, most farmers commonly used TCs for treatment of cattle, finding low residue levels may imply that there was no recent use of the antibiotic. Nevertheless, this is good news because the raw milk supplied in Bukombe district contain antibiotic residues at low levels than recommended MRL. Differently, Ridhiwan (2015) and Kimambo (2024) reported 10% and 2.5% of the milk samples from dairy cattle respectively to have tetracyclines residues above MRL in Tanzania. During the current study, the 54 farmers admitted accessing the medicines from the veterinary shops and livestock markets without any restrictions. These practices fuel the indiscriminate uses and consequently lead to antimicrobial residues, antimicrobial resistance and other health effects (Katakweba et al. 2012; Mdegela et al. 2021; Sangeda et al. 2021). The Veterinary Act Cap 319 (2003) and The Tanzania Food, Drugs and Cosmetics Act Cap 219 (2003) restrict haphazard sales of veterinary drugs, but the problem has been on enforcement especially in rural areas. A continuous education to farmers on the use veterinarians in health management of livestock is insisted. Law enforcement to curb uncontrolled sales of veterinary drugs is of paramount important.

### 4.1 Limitation of the Study

This study was a cross‐sectional design that involved collection of blood samples for brucellosis analysis and milk samples for antimicrobial analysis in one out of 184 district councils of Tanzania. Therefore, although the results represent the general picture of real situation on brucellosis and antimicrobial uses in pastoral and agropastoral communities of Tanzania, and East Africa, some more information may be missing. Further, tetracycline residues were identified and quantified only in 10 out 23 milk samples that were qualitatively positive for antimicrobial residues because of cost limitations. The unanalysed samples might have detectable levels of tetracycline residues that would give higher number of samples with significant tetracycline residues. Additionally, the study used only three seropositive samples to confirm *B. abortus* by PCR which is a small sample size to warrant for generality of results on the Bukombe District (see Supporting Information).

## 5 Conclusion

Based on the results of the current study, it is concluded that most farmers either neglect or are unaware of drug withdrawal periods which could be the reason for detection of antimicrobial residues in raw milk. It is insisted that Veterinary Council of Tanzania and Tanzania Medicines and Medical Devices Authority (TMDA) should enforce legislations to combat haphazard sales and uses of veterinary medicines. Bovine brucellosis is prevalent in Bukombe district. Good livestock husbandry practices and implementation of mandatory vaccination against brucellosis are recommended. Further, a coordinated One Health approach in the control of this public health threats is important to ensure healthy cattle, safe milk, protect consumer health, and preserving the effectiveness of antibiotics.

## Author Contributions

Conceptualising of research idea, writing of proposal, data collection and drafting of manuscript: M.M. Supervision of the whole research, data analysis and interpretation, review and perfection of manuscript: H.E.N. Both authors have read and agreed to the published version of the manuscript.

## Funding

The research work was co‐funded by Mwalimu Julius K. Nyerere University of Agriculture and Technology (MJNUAT) and One Health Organization for Central and East Africa (OHCEA).

## Ethics Statement

To observe animal welfare and rights, study animals were physically restrained using a halter to avoid fear, distress, physical discomfort, pain, and injury. The research was conducted according to the guidelines provided by the code of ethics of Sokoine University of Agriculture. Research permit No. SUA/ADM/R.1/8 was provided by the Vice Chancellor of Sokoine University of Agriculture on behalf of the Commission of Science and Technology (COSTECH). Permission number BDC/V.20/9/70 was provided by Bukombe District Executive Director and every time, the District Veterinary Officer accompanied the researchers to make sure that animal welfare and rights are fulfilled during handling of animals. Verbal consent was requested from each of the study farmers. Participation in the study was by voluntary basis. All the data that was collected from the respondents and the laboratory sample analysis was a confidential to the researcher.

## Conflicts of Interest

The authors declare that they have no conflicts of interest.

## Clinical Study Registration Number

None since this study did not involve clinical trials.

## Supporting information




**Supporting File**: vms370944‐sup‐0001‐SuppMat.doc

## Data Availability

The data generated through this study are contained in the manuscript. Additional data sets are available upon reasonable request.

## References

[vms370944-bib-0001] Aalipour, F. , M. Mirlohi , and M. Jalali . 2013. “Prevalence of Antibiotic Residues in Commercial Milk and Its Variation by Season and Thermal Processing Methods.” International Journal of Environmental Health Engineering 2: 41.

[vms370944-bib-0002] Addo, K. K. , G. I. Mensah , K. G. Aning , et al. 2011. “Microbiological Quality and Antibiotic Residues in Informally Marketed Raw Cow Milk Within the Coastal Savannah Zone of Ghana.” Tropical Medicine and International Health 16: 227–232. 10.1111/j.1365-3156.2010.02666.x 21070512

[vms370944-bib-0003] Adesokan, H. K. , P. I. Alabi , and M. A. Ogundipe . 2016. “Prevalence and Predictors of Risk Factors for Brucellosis Transmission by Meat Handlers and Traditional Healers' Risk Practices in Ibadan, Nigeria.” Journal of Preventive Medicine and Hygiene 57: E164–E171. PMC5139612 27980381

[vms370944-bib-0004] Ahlberg, S. , H. Korhonen , E. Lindfors , and E. Kang'ethe . 2016. “Analysis of Antibiotic Residues in Milk From Smallholder Farms in Kenya.” African Journal of Dairy Farm and Milk Production 3, no. 4: 152–158.

[vms370944-bib-0005] AOAC International . 2000. “AOAC Official Method 995.09 Chlortetracycline, Oxytetracycline and Tetracycline in Edible Animal Tissues: Liquid Chromatographic Method.” http://down.40777.cn/standard/8/23.1.17%20AOAC%20Official%20Method%20995.09%20/Chlortetracycline.pdf. 8920127

[vms370944-bib-0006] Applegren, L. , D. Arnold , J. Boisseau , et al. 1999. “Evaluation of Certain Veterinary Drug Residues in Food.” Fiftieth Report of the Joint FAO/WHO Expert Committee on Food Additives 888: 1–95. 10416362

[vms370944-bib-0007] Assenga, J. A. , L. E. Matemba , S. K. Muller , J. J. Malakalinga , and R. R. Kazwala . 2015. “Epidemiology of *Brucella* Infection in the Human, Livestock and Wildlife Interface in the Katavi‐Rukwa Ecosystem, Tanzania.” BMC Veterinary Research 11: 189. 10.1186/s12917-015-0504-8 PMC4529704 26253151

[vms370944-bib-0008] BDC Report . 2020. “Bukombe District Council Annual Report of 2020.” https://bukombedc.go.tz/.

[vms370944-bib-0009] Bodenham, R. F. , A. S. Lukambagire , R. T. Ashford , et al. 2020. “Prevalence and Speciation of Brucellosis in Febrile Patients From a Pastoralist Community of Tanzania.” Scientific Reports 10: 7081. 10.1038/s41598-020-62849-4 PMC7184621 32341414

[vms370944-bib-0010] Bukuku, J. N. , H. E. Nonga , and M. A. M. Mtambo . 2015. “Assessment of Raw Milk Quality and Stakeholders' Awareness on Milk‐Borne Health Risks in Arusha City and Meru District, Tanzania.” Tropical Animal Health Production 47, no. 5: 927–932. 10.1007/s11250-015-0810-y 25863955

[vms370944-bib-0011] CAC . 2023. “Maximum Residue Limits (MRLs) and Risk Management Recommendations (RMRs) for Residues of Veterinary Drugs in Foods CX/MRL, 2–2023.” http://www.fao.org/fao‐who‐codexalimentarius.

[vms370944-bib-0012] Canon, R.M. and Roe, R.T . 1986. Livestock Disease Surveys: A Field Manual for Veterinarians, 2nd ed. Queanbeyan, N.S.W., Australia: Better Printing Services.

[vms370944-bib-0013] Capparelli, R. , M. Parlato , M. Iannaccone , et al. 2009. “Heterogeneous Shedding of *Brucella Abortus* in Milk and Its Effect on the Control of Animal Brucellosis.” Journal of Applied Microbiology 106: 2041–2047. 10.1111/j.1365-2672.2009.04177.x 19298512

[vms370944-bib-0014] Cash‐Goldwasser, S. , M. J. Maze , M. P. Rubach , et al. 2018. “Risk Factors for Human Brucellosis in Northern Tanzania.” American Journal of Tropical Medicine and Hygiene 98: 598–606. 10.4269/ajtmh.17-0125 PMC5929176 29231152

[vms370944-bib-0015] Chitupila, G. Y. , E. V. G. Komba , and N. J. Mtui‐Malamsha . 2015. “Epidemiological Study of Bovine Brucellosis in Indigenous Cattle Population in Kibondo and Kakonko Districts, Western Tanzania.” Livestock Research Rural Development 27, no. 6: 1–12.

[vms370944-bib-0016] Chota, A. C. , H. B. Magwisha , B. Stella , et al. 2016. “Prevalence of Brucellosis in Livestock and Incidences in Humans in East Africa.” African Crop Science Journal 24: 45–52.

[vms370944-bib-0017] Crump, J. A. , A. B. Morrissey , W. L. Nicholson , R. F. Massung , R. A. Stoddard et al. 2013. “Etiology of Severe Non‐Malaria Febrile Illness in Northern Tanzania: a Prospective Cohort Study.” PLOS Neglected Tropical Diseases 7, no. 7: e2324. 10.1371/journal.pntd.0002324 PMC3715424 23875053

[vms370944-bib-0018] Dey, S. , and M. H. Karimm . 2013. “Study on Physicochemical and Microbial Quality of Available Raw, Pasteurized and UHT Milk During Preservation.” International Journal of Science Inventory Today 2, no. 2: 150–157.

[vms370944-bib-0019] EU (European Commission) . 2009. “On Pharmacologically Active Substances and Their Classification Regarding Maximum Residue Limits in Foodstuffs of Animal Origin.” Commission Regulation (EU). No 37/2010 of 22 December 2009, 1–76.

[vms370944-bib-0020] Ghidini, S. , E. Zanardi , G. Varisco , and R. Chizzolini . 2003. “Residues of β‐Lactam Antibiotics in Bovine Milk: Confirmatory Analysis by Liquid Chromatography Tandem Mass Spectrometry After Microbial Assay Screening.” Food Additive and Contaminants 20: 528–534. 10.1080/0265203031000098696 12881125

[vms370944-bib-0021] Grădinaru, A. C. , O. Popescu , and G. Solcan . 2011. “Antibiotic Residues in Milk From Moldavia, Romania.” Human and Veterinary Medicine 3, no. 2: 133–141.

[vms370944-bib-0022] Hesterberg, U. W. , R. Bagnallb , K. Perrettb , B. Boschb , R. Hornerb , and B. Gummow . 2008. “A Serological Prevalence Survey of *Brucella Abortus* in Cattle of Rural Communities in the Province of KwaZulu‐Natal, South Africa.” Journal of South African Veterinary Association 79, no. 1: 15–18. 10.4102/jsava.v79i1.234 18678186

[vms370944-bib-0023] Karimuribo, E. D. , P. L. Gallet , N. H. Ng'umbi , et al. 2015. “Status and Factors Affecting Milk Quality Along the Milk Value Chain: A Case of Kilosa District, Tanzania.” *Livestock Research for Rural Development* *27*: Article #51. Retrieved April 5, 2026. http://www.lrrd.org/lrrd27/3/kari27051.html.

[vms370944-bib-0024] Karimuribo, E. D. , L. J. Kusiluka , R. H. Mdegela , A. M. Kapaga , C. Sindato , and D. M. Kambarage . 2005. “Studies on Mastitis, Milk Quality and Health Risks Associated With Consumption of Milk From Pastoral Herds in Dodoma and Morogoro Regions, Tanzania.” Journal of Veterinary Science 6, no. 3: 213–221. 16131824

[vms370944-bib-0025] Karimuribo, E. D. , H. A. Ngowi , E. S. Swai , and D. M. Kambarage . 2007. “Prevalence of Brucellosis in Crossbred and Indigenous Cattle in Tanzania.” *Livestock Research for Rural Development* *19*: Article #148. Retrieved April 5, 2026. http://www.lrrd.org/lrrd19/10/kari19148.htm.

[vms370944-bib-0026] Katakweba, A. A. S. , M. M. A. Mtambo , J. E. Olsen , and A. P. Muhairwa . 2012. “Awareness of Human Health Risks Associated With the Use of Antibiotics Among Livestock Keepers and Factors That Contribute to Selection of Antibiotic Resistance Bacteria Within Livestock in Tanzania.” *Livestock Research for Rural Development* *24*: Article #170. Retrieved April 5, 2026. http://www.lrrd.org/lrrd24/10/kata24170.htm.

[vms370944-bib-0027] Kimambo, J. 2024. “Assessment of Antibiotic Use in Dairy Farming and Tetracycline and Sulphonamide Residues in Fresh Milk in Coast Region in Eastern Tanzania.” A Dissertation for Degree of Master of Public Health of Muhimbili University of Health and Allied Sciences, Dar es Salaam, Tanzania, 88.

[vms370944-bib-0028] Kivaria, F. M. , J. P. Noordhuizen , and A. M. Kapaga . 2006. “Evaluation of the Hygienic Quality and Associated Public Health Hazards of Raw Milk Marketed by Smallholder Dairy Producers in the Dar es Salaam Region, Tanzania.” Tropical Animal Health Production 38, no. 3: 185–194. 10.1007/s11250-006-4339-y 16986766

[vms370944-bib-0029] Landis, J. R. , and G. G. Koch . 1997. “The Measurement of Observer Agreement for Categorical Data.” Biometrics 33, no. 1: 159–174. 843571

[vms370944-bib-0030] Lyimo, B. E. 2013. “Prevalence of Bovine Brucellosis in Smallholder Dairy Farms in Morogoro Municipality, Tanzania.” A Dissertation for the Degree of Master of Science in Public Health and Food Safety of Sokoine University of Agriculture Morogoro, Tanzania, 86.

[vms370944-bib-0031] Mangsi, A. S. , M. Khaskheli , A. H. Soomro , and M. G. Shah . 2014. “Detection of Antimicrobial Drug Residues in Milk Marketed at Different Areas of Sindh Province.” IOSR Journal of Agriculture and Veterinary Science 7, no. 5: 65–69.

[vms370944-bib-0032] Mdegela, R. H. , E. R. Mwakapeje , B. Rubegwa , et al. 2021. “Antimicrobial Use, Residues, Resistance and Governance in the Food and Agriculture Sectors, Tanzania.” Antibiotics 10: 454. 10.3390/antibiotics10040454 PMC8073917 33923689

[vms370944-bib-0033] Mdegela, R. H. , R. Ryoba , E. D. Karimuribo , et al. 2009. “Prevalence of Clinical and Subclinical Mastitis and Quality of Milk in Smallholder Dairy Farms in Tanzania.” Journal of South African Veterinary Association 80, no. 3: 163–168. 10.4102/jsava.v80i3.195 20169749

[vms370944-bib-0034] Mekuria, S. , A. Regassa , R. Abebe , A. Fekade , and B. Dares . 2014. “Bacteriological Study Collected From Hawassa Smallhlder Dairy Farms.” Advances in Biological Research 8, no. 5: 194–200.

[vms370944-bib-0035] Mellau, L. S. B. , S. L. Kuya , and P. N. Wambura . 2009. “Seroprevalence of Brucellosis in Domestic Ruminants in Livestock‐Wildlife Interface: a Case Study of Ngorongoro Conservation Area, Arusha, Tanzania.” Tanzania Veterinary Journal 26, no. 1: 44–50.

[vms370944-bib-0036] Mengele, I., J. , G. M. Shirima , B. M. Bronsvoort , L. E. Hernandez‐Castro , and E. A. J. Cook . 2023. “Diagnostic Challenges of Brucellosis in Humans and Livestock in Tanzania: A Thematic Review.” CABI One Health 10: 1–16.

[vms370944-bib-0037] MLF . 2013. “Ministry of Livestock and Fisheries Budget Speech.” https://www.mifugouvuvi.go.tz/.

[vms370944-bib-0038] MLF . 2025. “Ministry of Livestock and Fisheries Budget Speech.” https://www.mifugouvuvi.go.tz/.

[vms370944-bib-0039] National Livestock Policy . 2006. “Ministry of Livestock and Fisheries, Tanzania.” https://www.mifugouvuvi.go.tz/publications/45.

[vms370944-bib-0040] Navarro, E. , J. Escribano , J. Fernandez .A, and J. Solera . 2002. “Comparison of Three Different PCR Methods for Detection of *Brucella* spp. in Human Blood Samples.” FEMS Immunology and Medical Microbiology 34: 147–151. 10.1111/j.1574-695X.2002.tb00616.x 12381466

[vms370944-bib-0041] Nielsen, K. , and D. Ewalt . 2010. “Bovine Brucellosis.” In VincenzoCaporale, Director General, OIE Biological Standards Commissio, Manual of Diagnostic Tests and Vaccines for Terrestrial Animals, 642–660. OIE.

[vms370944-bib-0042] OIE (Office International des epizooties) . 2012. “Bovine Brucellosis.” In Vincenzo Caporale, Director General, OIE Biological Standards Commission, Manual of Diagnostic Tests and Vaccines for Terrestrial Animals. OIE http://www.oie.int/en/international‐standard‐setting/terrestrial‐manual/.

[vms370944-bib-0043] Orwa, J. D. , J. W. Matofari , P. S. Muliro , and P. Lamuka . 2017. “Assessment of Sulphonamides and Tetracyclines Antibiotic Residue Contaminants in Rural and Peri Urban Dairy Value Chains in Kenya.” International Journal of Food Contaminations 4: 5.

[vms370944-bib-0060] Ramadhani, R. 2015. “Assessment of Antibiotic Residues in Raw Cows' Milk Produced by Small Scale Dairy Farms in Bagamoyo District, Tanzania. A dissertation for the Degree of Master of Science Public Health and Food Safety of Sokoine University of Agriculture.” Morogoro, Tanzania. 91pp. https://www.suaire.sua.ac.tz/items/fcdd36ee-1a58-47f3-aa38-24a0a570f32c.

[vms370944-bib-0044] Ridhiwani, R. 2015. “Assessment of Antibiotic Residues in Raw Cows' Milk Produced by Small Scale Dairy Farms in Bagamoyo District, Tanzania.” A Dissertation for the Degree of Master of Science Public Health and Food Safety of Sokoine University of Agriculture. Morogoro, Tanzania. 91 pp. https://www.suaire.sua.ac.tz/items/fcdd36ee‐1a58‐47f3‐aa38‐24a0a570f32c.

[vms370944-bib-0045] Rwehumbiza, J. M. , R. Ryoba , and E. D. Karimuribo . 2012. “Assessment of Microbiological Status and Presence of Antibiotic Residues in Cow Milk Produced in Bagamoyo and Kisarawe Districts, Tanzania.” Tanzania Veterinary Journal 28: 60–69.

[vms370944-bib-0046] Salman, A. M. , H. A. ElNasri , and I. A. M. Osman . 2012. “Detection of Antibiotic Residues in Milk Using Delvotest Kit and the Disc Assay Methods in Khartoum State, Sudan.” Journal of Veterinary Medicine and Animal Production 3, no. 2: 3–15.

[vms370944-bib-0047] Sangeda, R. Z. , A. Baha , A. Erick , et al. 2021. “Consumption Trends of Antibiotics for Veterinary Use in Tanzania: A Longitudinal Retrospective Survey From 2010–2017.” Frontiers in Tropical Diseases 2: 1–11.

[vms370944-bib-0048] Swai, E. S. , E. D. Karimuribo , L. Schooman , et al. 2005. “Description, Social‐Economic Characteristics, Disease Management and Mortality Dynamics in Smallholder's Dairy Production System in Coastal Humid Region of Tanga, Tanzania.” *Livestock Research for Rural Development* *17*: Article #41. Retrieved April 5, 2026. http://www.lrrd.org/lrrd17/4/swa17041.htm.

[vms370944-bib-0049] Tadesse, G. 2016. “Brucellosis Seropositivity in Animals and Humans in Ethiopia: A Meta‐Analysis.” PLOS Neglected Tropical Diseases 10, no. 10: e0005006. 10.1371/journal.pntd.0005006 PMC5085315 27792776

[vms370944-bib-0050] Temba, P. B. , R. S. Mwakapuja , Z. E. Makondo , et al. 2019. “Spatial Distribution and Risk Factors for Brucellosis in Domestic and Wild Animals at Livestock‐Wildlife Interface in Mikumi‐Selous Ecosystem, Tanzania.” Tanzania Veterinary Journal 34: 1–8.

[vms370944-bib-0051] The Animal Diseases Act Cap 156 . 2003. https://www.mifugouvuvi.go.tz/.

[vms370944-bib-0052] The Tanzania Food Drugs and Cosmetics Act . 2003. https://www.tmda.go.tz/.

[vms370944-bib-0053] The Veterinary Act Cap 319 . 2003. https://www.mifugouvuvi.go.tz/.

[vms370944-bib-0054] TLMP . 2017. “Tanzania Livestock Master Plan of 2017/2018 –2021/2022.” https://www.mifugouvuvi.go.tz/.

[vms370944-bib-0055] Tumwine, G. , E. Matovu , J. D. Kabasa , D. O. Owiny , and S. Majalija . 2015. “Human Brucellosis: Sero‐Prevalence and Associated Risk Factors in Agro‐Pastoral Communities of Kiboga District, Central Uganda.” BMC Public Health [Electronic Resource] 15: 900. 10.1186/s12889-015-2242-z PMC4572625 26374402

[vms370944-bib-0056] USA: CLG‐TET2.04 . 2011. “United States Department of Agriculture Food Safety and Inspection Service, Office of Public Health Science,” 13. USDA.

[vms370944-bib-0057] Wareth, G. , F. Melzer , M. C. Elschner , H. Neubauer , and U. Rosler . 2014. “Detection of *Brucella Melitensis* in Bovine Milk and Milk Products From Apparently Healthy Animals in Egypt by Real‐Time PCR.” Journal of Infection in Developing Countries 8, no. 10: 1339–1343. 10.3855/jidc.4847 25313613

[vms370944-bib-0058] Weinhäupl, I. , K. C. Schöpf , D. Khaschab , A. M. Kapaga , and H. M. Msami . 2000. “Investigations on the Prevalence of Bovine Tuberculosis and Brucellosis in Dairy Cattle in Dar es Salaam Region and in Zebu Cattle in Lugoba Area, Tanzania.” Tropical Animal Health and Production 32, no. 3: 147–154. 10.1023/a:1005231514467 10907285

[vms370944-bib-0059] World Bank . 2024. “Tanzania Economic Update: Harnessing the Opportunity for a Climate Smart and Competitiveness Livestock Sector in Tanzania.” World Bank Group. http://documents.worldbank.org/curated/en/099061224074521849/P1796101d6aa520c1b91b176e38367ab07.

