# Correction to “Occurrence and Public Health Implications of *Brucella Abortus* and Antimicrobial Residues in Raw Cow Milk in Bukombe District, Tanzania”

**DOI:** 10.1002/vms3.71201

**Published:** 2026-10-12

Mhozya, M., M. Julius K, and H. E. Nonga. 2026. “Occurrence and Public Health Implications of Brucella Abortus and Antimicrobial Residues in Raw Cow Milk in Bukombe District, Tanzania.” *Veterinary Medicine and Science* 12, no. 3: e70944. https://doi.org/10.1002/vms3.70944.

The name Mwalimu Julius K was inadvertently included in the author list. As it refers to the institution of the first author, Mhozya Makoye, rather than an author, it has been removed from the author list.

The online original article is also corrected.

We apologize for this error.

