# Supplementary material for: Occurrence and Public Health Implications of Brucella Abortus and Antimicrobial Residues in Raw Cow Milk in Bukombe District, Tanzania
Source: Vet Med Sci. 2026 Apr 9;12(3):e70944. doi: 10.1002/vms3.70944 (PMC13063390; doi:10.1002/vms3.70944)
Supplement: Supplementary file 1 — Supporting File: vms370944‐sup‐0001‐SuppMat.doc [file VMS3-12-e70944-s001.doc]

**SUPPLEMENTARY MATERIALS**

**1.0 Analysis of antimicrobial residues in milk by chromatographic method**

Analysis for antimicrobial residues in milk was done using High Performance Liquid Chromatography (HPLC). All the reagents and chemicals were of HPLC or analytical grade. The reagents included tetracyclines CRS standard (Sigma, St Louis), oxalic acid dihydrate, citric acid monohydrate and disodium ethylenediaminetetraacetate (Na_2_EDTA), ammonium hydroxide (Fishers Scientific Limited, UK), anhydrous disodium hydrogen phosphate (Carlo Ebra, Milan), methanol, acetonitrile and HPLC water (Carlo Ebra, Milan). Whatman membranes, microsyringe membrane filters (Chromafil CA 20/25s), GFB filter papers, nylon membranes (P/N 0235-0301) and Cronus C-18 solid-phase extraction cartridges (200 mg/3 mL, Labhut) were used for the chromatography steps.

Analysis was carried out on a HPLC Model LC-20AT Prominence Liquid Chromatography series (SHIMADZU Corporation, Japan.), equipped with constant flow pump, degasser (DGU 20A_5_ Serial L-20244 07326), column oven (CTO-10A VP Serial C 210447 06967), auto sampler (OPTIMAS- Spark) and computer software. Separations were conducted by BD5 Hypersil-Analytical C8 (125x 4 mm, particle size: 5 μm, part number: 28205-124030 and serial number: 10058200, Column Agilent Technologies- USA). Analyte was detected by PDA-Detector with variable wavelength UV detector (SPD-20A), CBM- 20A Prominence serial No L 202347 09807 (SHIMADZU Corporation Japan). Oxalic acid- Acetonitrile- Solution in a ratio of 75:25 was used as a liquid chromatography mobile phase.

**1.1 Sample preparation, extraction and cleaning up**

Homogenised, previously frozen raw milk samples (5.0 ±0.1 ml test milk), showing no signs of souring or curdling was pipetted into 50 ml polypropylene centrifuge tube and centrifuged for 10 minutes at 4000 rpm at approximately 15°C temperature to separate the cream. The contents were then mixed with 20 ml Mcllvaine buffer/EDTA solution and shaken for 10 minutes on flat bed shaker at high speed. The content of the tubes was then centrifuged for 10 minutes at 4000 rpm at approximately 15°C. The supernatant was filtered through GFB filter paper moistened with Mcllvaine buffer-EDTA solution. The sample clean up procedure was done by solid phase extraction (C18 SPE) cartridges that were attached to an SPE vacuum manifold connected to 75 ml reservoir to each cartridge. The Bond Elut-SAX C18 cartridges were first conditioned with 10 ml methanol followed by 20 ml of HPLC- grade water, at 1.5-2.5 ml/minute, vacuum was applied gently, and the eluate was discarded.

**1.2 High performance liquid chromatographic analysis for tetracyclines**

By using 0.45 μm pore size microsyringe filter, the test solution and the standards were filtered into the LC auto sampler vials and loaded into auto sampler. The analysis and quantification of the TCs residue in the extracts was done using a high performance liquid chromatography (LC-20AT Model Shimadzu Prominence series -Japan) equipped with CBM-20A auto injector with sample cooler CTO-10AS VP and DGU-20A_5_ on-line vacuum degassing solvent delivery unit, a constant flow pump and a variation wavelength UV detector set at 365 nm. The separation was done on BD5 Hypersil-analytical C8 (125x 4 mm, particle size: 5 µ) column with oxalic acid-acetonitrile solution (75:25) as the mobile phase by gradient mode, the mobile phase flow-rate was 1.2 ml/min at room temperature and the sensitivity range was 0.08 ppm. The HPLC analysis was performed for 7 minutes in each sample.

To determine residues in the samples, analysis was done concurrently with the oxtetracycline standard solutions (25 μg/mL, 50 μg/mL, 75 μg/mL, 100 μg/mL, 125 μg/mL, 150 μg/mL and 200 μg/mL). The extract from each sample was injected in duplicate to obtain an average peak height of positive samples. The samples were positive for tetracyclines (TCs) if the retention time and peaks corresponded to those of the reference standards. The retention time of the standards were at 2.9, 3.4, 5.6 min for oxytetracycline (OTC), tetracycline (TTC) and Chlortetracycline (CTC), respectively.

Table 1. OTC standard calibration curve

| Point | Concentration (µg/l) | Mean Area | Area |
| --- | --- | --- | --- |
| 1 | 25.0 | 23293.3 | 23293 |
| 2 | 50.0 | 68095.7 | 68096 |
| 3 | 75.0 | 91907.1 | 91907 |
| 4 | 100.0 | 155503.0 | 155503 |
| 5 | 125.0 | 189912.2 | 189912 |
| 6 | 150.0 | 268661.7 | 268662 |
| 7 | 200.0 | 324666.4 | 324666 |

Figure 1. Calibration curve with seven concentration points of OTC standard

Table 2. TTC standard calibration curve

| Point | Concentration (µg/l) | Mean Area | Area |
| --- | --- | --- | --- |
| 1 | 25.0 | 24385.4 | 24385 |
| 2 | 50.0 | 76911.6 | 76912 |
| 3 | 75.0 | 101337.0 | 101337 |
| 4 | 100.0 | 169609.7 | 169610 |
| 5 | 125.0 | 213167.3 | 213167 |
| 6 | 150.0 | 288528.1 | 288528 |
| 7 | 200.0 | 343180.6 | 343181 |

Figure 2. Calibration curve with seven concentration points of TTC standard

Table 3: CTC standard calibration curve

| Point | Concentration (µg/l) | Mean Area | Area |
| --- | --- | --- | --- |
| 1 | 25.0 | 16421.9 | 16422 |
| 2 | 50.0 | 47882.3 | 47882 |
| 3 | 75.0 | 72664.9 | 72665 |
| 4 | 100.0 | 117798.8 | 117799 |
| 5 | 125.0 | 146613.3 | 146613 |
| 6 | 150.0 | 179097.1 | 179097 |
| 7 | 200.0 | 237460.2 | 237460 |

Figure 3. Calibration curve with seven concentration points of CTC standard

**1.3 Method development for HPLC analysis of raw milk samples**

Prior to the extraction of TCs in an unknown raw milk sample, the method was developed. Under gradient conditions, different standard concentrations of the prepared TCs standard solutions were injected in ascending order and the results were plotted automatically on the integrator (Figure 4). The machine was set to run for 7 minutes and TCs were detected at an average of 2.9, 3.4, 5.6 min as retention time of OTC, TTC, CTC respectively with the peak height increasing with increase in concentrations.

Figure 4. The chromatogram of mixed standard solution of TCs (104. 1 µg/l)

**1.4 Control samples**

Two hundred microliter (200 μl) of the working solution of TCs standard (25 μg/ml) was pipetted into 5 ml volumetric flask, followed by addition of antibiotic free UHT milk and shake thoroughly for 10 seconds to mix and left for 30 minutes before starting extraction procedures. The UHT milk was subjected separately to the extraction, clean-up and elution procedures as described for the test milk samples derived from lactating cows. The retention time of the control samples was compared with those of the TCs standard solution alone. A blank sample eluted from the solid-phase extraction cartridge was included to check for the analytical column efficiency during extraction. The recoveries of the TCs were calculated at six different concentration levels (25 μg/mL, 50 μg/mL, 100 μg/mL, 125 μg/mL, 150 μg/mL, 200 μg/mL). The recovery rates were displayed at averages of 86.6%, 86.5% and 84.4% for OTC, TC and CTC, respectively. The mean recovery of the TCs was 85.8% (Table 4).

Table 4. Recovery and precision of TCs determined in spiked UHT milk sample (n=6)

| Fortification level (µg/l) | OTC (%) | TTC (%) | CTC (%) | Mean recovery (%) |
| --- | --- | --- | --- | --- |
| 25 | 87.7 | 90.8 | 90.3 | 89.6 |
| 50 | 88.9 | 94.1 | 92.6 | 91.9 |
| 100 | 88.8 | 86.3 | 81.1 | 85.4 |
| 125 | 84.1 | 88.1 | 79.8 | 83.9 |
| 150 | 82.6 | 80.3 | 81.1 | 81.3 |
| 200 | 87.3 | 79.3 | 81.5 | 82.7 |
| Mean recovery (%) | 86.6 | 86.5 | 84.4 | 85.8 |

The Mean recovery rate of the Tetracyclines was 85.8%
